# Life-course blood pressure trajectories and incident diabetes: A longitudinal cohort in a Chinese population

**DOI:** 10.3389/fendo.2022.1035890

**Published:** 2022-11-11

**Authors:** Ying Liu, Xiaohong Chen, Chunxia Li, Bingbing Fan, Jiali Lv, Yanlin Qu, Yongjiang Cai, Tao Zhang

**Affiliations:** ^1^ Department of Biostatistics, School of Public Health, Cheeloo College of Medicine, Shandong University, Jinan, Shandong, China; ^2^ Institute for Medical Dataology, Shandong University, Jinan, China; ^3^ Center of Health Management, Beijing University Shenzhen Hospital, Shenzhen, Guangdong, China

**Keywords:** systolic blood pressure, diabetes, prediabetes, trajectory, longitudinal study

## Abstract

**Background:**

Blood pressure levels are correlated with diabetes among middle-aged or older adults. However, longitudinal trajectories of blood pressure during young adulthood and their impact on diabetes have been insufficiently studied.

**Methods:**

The longitudinal cohort consisted of 4,625 adults who had blood pressure and body mass index (BMI) repeatedly measured five to nine times during 18–60 years of age. Distinct systolic blood pressure (SBP) trajectories were identified by a group-based trajectory model. Logistic regression analyses were used to investigate the association between trajectory patterns or quartiles of area under the curve values of SBP trajectories and incident diabetes, respectively.

**Results:**

Four distinct trajectory groups were identified for SBP: normotensive-stable (n = 761, 16.5%), prehypertension-stable (n = 2,381, 51.5%), stage I hypertension-increasing (n = 1,231, 26.6%), and stage II hypertension-increasing (n = 251, 5.4%). Compared with subjects who remained at SBP <120 mmHg in the normotensive-stable group, individuals in the prehypertension-stable trajectory exhibited a normal SBP range (<140 mmHg), and they still had a significantly higher risk of diabetes (adjusted OR = 1.82, p = 0.029). Individuals had a greater risk of diabetes in the stage I hypertension-increasing group (adjusted OR = 2.31, p = 0.006) and the highest risk in the stage II hypertension-increasing group (adjusted OR = 3.91, p < 0.001) relative to the normotensive-stable group. Furthermore, compared with the first quartile, adjusted ORs (95% CIs) of the fourth quartile of SBP incremental and total AUC were 2.50 (1.61–3.97) and 1.82 (1.15–2.94), respectively.

**Conclusions:**

Long-term SBP trajectory is a significant predictor for incident diabetes, which is independent of baseline SBP and body weight, attaching importance to maintaining optimal blood pressure levels and controlling changing slopes of SBP for preventing diabetes.

## Introduction

Diabetes has emerged as one of the most serious and common chronic diseases (1). The global prevalence of diabetes has increased significantly in recent decades (2–6), and it was reported to be 10.5% among adults (aged 20–79 years), affecting 536.6 million adults in 2021 (7). The prevalence of diabetes in China has increased from 0.67% in 1980 (8) to 12.4% in 2018 (9). It is particularly important to identify patients at high risk for diabetes for developing effective preventive measures (10, 11). Numerous clinical and epidemiologic studies have demonstrated that type 2 diabetes mellitus (T2DM) is correlated with obesity and blood pressure (BP) (12–17). In addition to the absolute level of these risk factors, increasing evidence indicates that their longitudinal growth trajectory also plays a considerable role in the incident T2DM (18–22). However, most previous studies focused on the body mass index (BMI) and/or systolic BP in a single measurement or only paid attention to the association between the trajectories of BMI and incident diabetes, ignoring the dynamic trends of SBP in the life course.

Diabetes and hypertension are chronic medical conditions that frequently coexist (23). In 2019, the global age-standardized prevalence of hypertension in adults (aged 30–79 years) was 32%, which was similar to that in 1990 but with the number of hypertension doubled (24). Among Chinese adults aged 35–75 years, nearly one-third have hypertension (25). Baseline BP is a strong predictor of T2DM among initially healthy subjects, and this effect is independent of BMI and other metabolic syndrome components (13–15, 26). Although numerous studies have demonstrated the association between BP measurements and incident diabetes, a single BP measurement may be insufficient to predict the long-term risk of incident diabetes. The change in BP over time is an important risk factor, and the relationship between BP trajectories and mid-life adulthood diabetes should be considered (27). There were a few studies that reported the longitudinal profiles of BP across the life course and their impact on cardiovascular disease (CVD) or mortality (28–30). However, the association between BP trajectories and incident diabetes has been insufficiently studied to the best of our knowledge, and this relationship remains to be explored.

Although the progression of SBP with age for most individuals is a well-known phenomenon, the growth patterns of SBP vary considerably because of population heterogeneity. It still remains unclear whether the dynamic trends of SBP over time would affect incident diabetes in later life. Long-term trajectory assessment has been used to reflect SBP growth patterns. We also hypothesized that participants who experience higher SBP levels and faster rates of increase in SBP would have a higher diabetes risk compared with the trajectory group in which individuals remain on ideal SBP levels in their life course. In addition to the absolute levels of SBP, the longitudinal SBP trajectories over the life course also play a considerable part in developing diabetes.

The main aim is to identify distinct SBP trajectory profiles from early to mid-life adulthood (18–60 years old) using data from the China Health and Nutrition Survey (CHNS) and to explore the association of SBP trajectories with incident diabetes. The SBP trajectories would identify more individuals at high risk for developing diabetes and help clinicians become aware of these relationships to optimize the management of subjects with dynamic rises in SBP.

## Methods

### Study population

The CHNS is an ongoing prospective cohort study, and it is the only large-scale longitudinal, household-based survey in China. Ten cross-sectional surveys were completed between 1989 and 2015. In the current survey, multistage, random cluster sampling was used to sample approximately 11,130 households with about 40,000 individuals (31, 32). This present study sample included nine surveys undertaken between 1989 and 2011. **Figure S1** shows the inclusion and exclusion process of participants in this study. We excluded participants if they met any of the following criteria (1): age <18 years or age >60 years (2); missing BMI and BP information; and (3) BMI or BP measurements <5 times (because of the requirement for trajectory analysis). Subjects without diabetes at baseline who were 18–60 years old and with more than four follow-up visits were eligible in the current trajectory analysis of SBP and BMI. Observations after individuals developed diabetes were all excluded.

The protocol was approved by the Ethics Committee of the National Institute for Nutrition and Health, China CDC (Number 201524). All study participants provided written informed consent. All procedures complied with the principles of the Declaration of Helsinki.

### General examinations

After a 10-min rest, three measurements of SBP and diastolic blood pressure (DBP) were taken on the right arm in the sitting position using a mercury sphygmomanometer, and the average of the three measurements was used in the analysis. Height and weight were measured in light clothing and without shoes to the nearest 0.1 cm and 0.1 kg, respectively, and they were all measured three times to calculate the mean value. BMI was calculated by dividing weight in kilograms by height in meters squared. Individuals were defined as smokers only if they had a history of smoking or currently smoke; those with past or current alcohol consumption were defined as drinkers (32).

Based on the nutrition profile designed for the 2002 CHNS, detailed food consumption information for three consecutive days translated into food groups was used for the dietary assessment (33). Physical activity includes light manual work (such as sedentary work, occasional standing and sitting work, office work, watchmakers, shop assistants, and laboratory technicians), moderate physical labor (such as drivers and electricians), and heavy manual labor (such as farmers, athletes, dancers, steelworkers, lumberjacks, and construction workers).

### Measurements

Diabetes was defined as one or more of the following (1): taking any treatment of diabetes (18) (2); fasting blood glucose ≥7.0 mmol/L and/or HbA1c ≥6.5%; and (3) self-reported diabetes (34, 35). The participants were asked to report the history of diabetes with a questionnaire-based interview during the follow-up, and the questions were posed as follows (1): has a doctor ever told you that you suffer from diabetes? and (2) how old were you when the doctor told you about such a situation (years)? Whole HbA1c and serum glucose were used to identify diabetes in the 2009 survey. Prediabetes was defined as fasting blood glucose of 5.6–6.9mmol/L and/or HbA1c of 5.7%–6.4%. Data on self-reported diabetes and glucose-lowering medication were first collected in 1997 and in subsequent surveys. Using fasting blood glucose and HbA1c identified incident diabetes and prediabetes in the 2009 survey. Hypertension was defined as one or more of the following (1): taking antihypertensive medication (2); SBP/DBP ≥140/90 mmHg; and (3) self-reported hypertension (36, 37). The participants were asked to report their previous history of hypertension with the following questions (1): has a doctor ever told you that you suffer from hypertension? and (2) how old were you when the doctor told you about such a situation (years)?

### Statistical analysis

Group-based trajectory models (GBTMs) were used to identify the latent growth trajectories of SBP. These models were fit by the Traj procedure in SAS (version 9.4). The Appendix in the Supplementary Material contains details of the trajectory modeling process (**Tables S1**, **S2**). We tested models using linear and non-linear polynomial function parameters with the number of groups changing from 2 to 5. The selection criteria of the optimal number of groups and growth curve shapes were reported in a previous study (38), including the lowest Bayesian information criteria value and high mean posterior class membership probabilities greater than 0.65. The sample size in each latent trajectory class should not be less than 5% of the total cohort. Each participant was assigned to the latent trajectory group that he/she is most likely to be in with the highest probability. Trajectory groups were data-derived and subsequently labeled based on the groups characterized by the latent class analysis. We also identified the latent growth trajectory of BMI in the same way.

The relationship between the SBP trajectory group membership and diabetes was examined by the logistic regression models without adjustments (Model 1); further with adjustments to gender, baseline age, SBP, BMI, and follow-up years (Model 2); additional adjustments to smoking, alcohol drinking, physical activity, energy intake, and antihypertensive drugs use (Model 3); and adjustments to BMI trajectory groups (Model 4). Similarly, the associations between the joint group of SBP and BMI trajectories and incident diabetes were explored by the logistic regression models as well.

The non-linear growth curve parameters of SBP and BMI were estimated by a random-effects mixed model, which can generate 4,625 different sets of curve parameters for all the study participants. These models were fit by the R package lme4 that all specified the functions of age, which centered on 41 years (the mean age of the cohort). The maximum likelihood method was used to accomplish the model fitting and estimation of fixed- and random-effects parameters for the individuals. The random-effects coefficient represents the difference between the deviations of an individual from the mean of the population. The selection of the most parsimonious model has been reported in our previous study (39). Quadratic curves were fitted for SBP and BMI.

The long-term burden of SBP was calculated as the area under the growth curve (AUC) including total, incremental, and baseline AUC for each subject during the follow-up period. A previous research study has reported the measures of the three AUC values (18) using logistic regression models to investigate the relationship between these AUC values and incident diabetes. Quartiles of the three AUC values mentioned above were calculated before the logistic regression analysis.

Model-estimated parameters and first derivatives were used to calculate the levels and linear slopes of SBP at each age point taking 1 year as the interval. Logistic regression models were used to examine the relationships of the estimated levels and linear slopes of SBP with incident diabetes, and their standardized odds ratios (ORs) were estimated at each age point. In order to avoid collinearity between the SBP level and the linear slope in the same model, regression residual analysis was performed for the estimated linear slope of SBP with adjustment for the covariables mentioned above, the model estimated level and the linear slope of BMI, and their corresponding SBP levels at the same age point.

The association between SBP trajectory patterns and incident prediabetes as well as the joint group of SBP and BMI trajectories and incident prediabetes was explored by the logistic regression models. Characteristics of different groups were evaluated using Student’s *t*-test or the one-way ANOVA test or the Wilcoxon rank sum test or the Kruskal–Wallis rank sum test for continuous variables, and the chi-square test for categorical variables. Two-tailed p-values less than 0.05 were considered statistically significant.

## Results

The baseline characteristics of participants by incident diabetes groups are shown in **Table 1**. During the follow-up period, 248 cases of incident diabetes were recognized. Diabetes had a higher BMI, SBP, DBP, carbohydrate intake, and incident hypertensives than normoglycemia. Compared with the included individuals, those excluded tended to be younger, have lower BMI and BP levels, and have higher smoking and drinking rates (**Table S3** in the Supplement). **Table S4** shows the follow-up characteristics of the study for incident diabetes.

**Table 1:** Baseline characteristics by incident diabetes at follow-up.

Variable	Total	Normoglycemia	Diabetes	p
n	4625	4377	248	
Age, years	32.6 (7.2)	32.6 (7.3)	33.3 (6.2)	0.162
Males, n (%)	2185 (47.2)	2061 (47.1)	124 (50.0)	0.407
BMI, kg/m^2^	21.7 (2.5)	21.6 (2.5)	23.2 (2.9)	<0.001
SBP, mm Hg	111.7 (13.2)	111.5 (13.1)	114.8 (14.1)	<0.001
DBP, mm Hg	73.1 (9.8)	73.0 (9.7)	75.4 (9.9)	<0.001
Smoker, n (%)	537 (11.6)	515 (11.8)	22 (8.9)	0.200
Drinker, n (%)	602 (13.0)	571 (13.0)	31 (12.5)	0.880
Hypertension, n (%)	152 (3.3)	138 (3.2)	14 (5.6)	0.050
Physical-activity				0.029
Light, n (%)	1260 (27.2)	1192 (27.2)	68 (27.4)	
Moderate, n (%)	1418 (30.7)	1325 (30.3)	93 (37.5)	
Heavy, n (%)	1947 (42.1)	1860 (42.5)	87 (35.1)	
Energy intake, kcal/d	2524.1 (501.3)	2520.8 (507.5)	2583.0 (371.0)	0.057
Carbohydrate intake, g/d	406.8 (84.6)	406.1 (85.0)	418.9 (77.6)	0.020
Fat intake, g/d	63.4 (29.1)	63.4 (29.7)	63.4 (14.7)	0.997
Protein intake, g/d	76.5 (31.8)	76.3 (32.5)	78.7 (13.4)	0.250
FPG*, mmol/L	5.8 (1.9)	5.1 (0.6)	7.7 (2.7)	<0.001
HbA1c*, %	5.9 (1.5)	5.5 (0.4)	7.1 (2.3)	<0.001
Follow up, years	17.8 (3.8)	17.7 (3.8)	18.1 (3.0)	0.197

Data are means ± SD, or n (%).

*Follow-up information.

BMI, body mass index; SBP, systolic blood pressure; DBP, diastolic blood pressure; FPG, fasting plasma glucose; Hb1Ac, Hemoglobin A1c.

**Figure 1:**
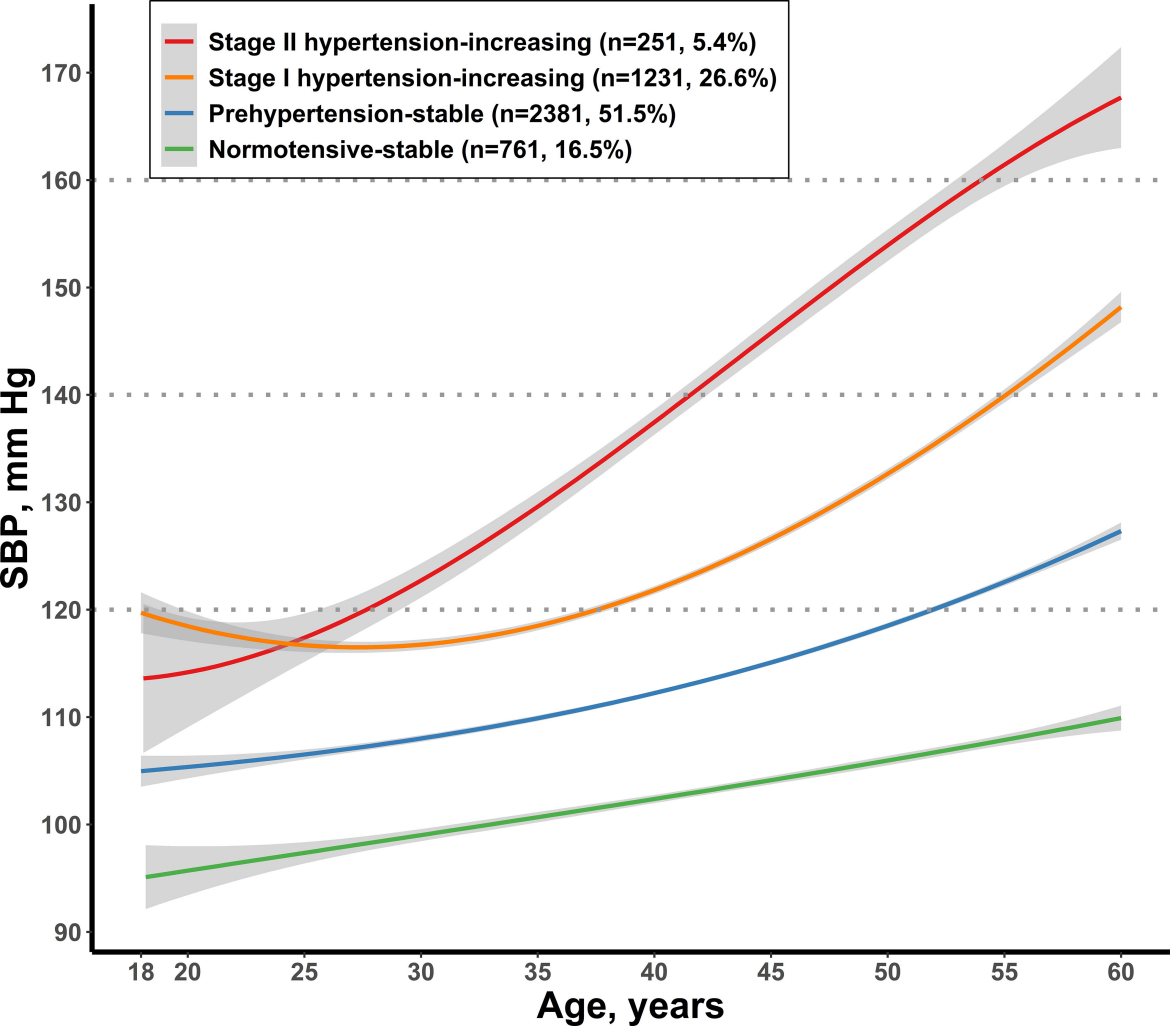
Trajectories of SBP during young adulthood. SBP, systolic blood pressure.

Four distinct trajectories of SBP from young adulthood to middle age among the 4,625 participants were identified (**Figure 1**). Each of the trajectories were labeled based on their SBP ranges and patterns over time: 761 participants (16.5%) had low SBP levels and remained at <120 mmHg throughout follow-up (normotensive-stable group); 2,381 (51.5%) had moderate levels of SBP that were relatively stable and remained at <140 mmHg throughout (prehypertension-stable group); 1,231 (26.6%) had the highest SBP of ≈120 mmHg at the age of 18 and then gradually increased to 140 mmHg at 55 years of age and after that maintaining <160 mmHg (stage I hypertension-increasing group); and 251 participants (5.4%) had higher SBP levels and increased rapidly throughout—increased to 140 mmHg at the age of 42 and then remained at <170 mmHg (stage II hypertension-increasing). **Table S5** presents the characteristics of participants by the SBP trajectory patterns. Diabetes and hypertension incidence, sex, BMI, SBP, DBP, smoking, alcohol drinking, and carbohydrate intake were significantly different among the four SBP trajectory groups.

**Figure S2** shows the three distinct trajectories of the included BMI: 2,343 participants (50.7%) had low BMI levels and remained at <24 kg/m^2^ throughout (referred to as normal-stable pattern); 1,332 (28.8%) had moderate BMI levels and increased to 24 kg/m^2^ at the age of 44 and after that maintaining <28 kg/m^2^ (referred to as overweight-increasing pattern); and 950 (20.5%) had higher BMI levels whose BMI increased to 24 kg/m^2^ at the age of 27 and increased to 28 kg/m^2^ at the age of 47 (referred to as obesity-increasing pattern).

**Table 2** shows the odds ratios (ORs) of SBP trajectory groups for incident diabetes. Even though individuals in the prehypertension-stable SBP trajectory group have maintained SBP levels in the normal range (<140 mmHg), they still had a significantly higher risk of diabetes (adjusted OR = 1.82) when compared with individuals in the normotensive-stable SBP pattern whose SBP remained throughout lower than 120 mmHg after adjusting for potential confounders (Model 3). With adjustment to BMI trajectory patterns, the diabetes risk associated with the prehypertension-stable SBP trajectory group was attenuated but remained significant (adjusted OR = 1.72, Model 4). Compared with the normotensive-stable SBP trajectory pattern, individuals had a higher risk of diabetes in the stage I hypertension-increasing group (adjusted OR = 2.31) and the highest risk in the stage II hypertension-increasing group (adjusted OR = 3.91) after adjustment for BMI trajectory patterns and other covariates in Model 4. There was no significant multiplicative gender interaction in the association between SBP trajectories and incident diabetes (interaction p = 0.649); thus, we conducted analyses by gender as well (**Table 2**). Men in the stage II hypertension-increasing SBP group had 4.69 times greater OR for incident diabetes compared with those in the normotensive-stable SBP group (Model 4); the corresponding OR for this association in women was 3.10 (p = 0.021).

**Table 2:** Odds ratios and 95% CIs for trajectories of SBP groups for incident diabetes.

	Model 1		Model 2		Model 3		Model 4	
	OR (95%*CI*)	1.1.1p	OR (95%*CI*)	1.1.2p	OR (95%*CI*)	1.1.3p	OR (95%*CI*)	p
**Total, n = 4625**								
Normotensive-stable	Ref		Ref		Ref		Ref	
Prehypertension-stable	2.02 (1.24, 3.52)	0.008	1.89 (1.13, 3.33)	0.020	1.82 (1.09, 3.21)	0.029	1.72 (1.03, 3.05)	0.049
Stage I hypertension-increasing	3.37 (2.04, 5.91)	<0.001	2.88 (1.64, 5.28)	<0.001	2.64 (1.49, 4.90)	0.001	2.31 (1.29, 4.30)	0.006
Stage II hypertension-increasing	7.82 (4.40, 14.47)	<0.001	5.72 (2.96, 11.37)	<0.001	4.82 (2.39, 9.94)	<0.001	3.91 (1.93, 8.13)	<0.001
**Males, n = 2185**								
Normotensive-stable	Ref		Ref		Ref		Ref	
Prehypertension-stable	2.03 (0.81, 6.79)	0.180	1.74 (0.68, 5.92)	0.301	1.68 (0.65, 5.72)	0.337	1.58 (0.61, 5.41)	0.397
Stage I hypertension-increasing	3.61 (1.46, 12.03)	0.014	2.76 (1.02, 9.67)	0.069	2.65 (0.97, 9.35)	0.083	2.28 (0.83, 8.10)	0.146
Stage II hypertension-increasing	9.32 (3.44, 32.59)	<0.001	6.37 (2.10, 24.02)	0.002	6.01 (1.91, 23.25)	0.004	4.69 (1.46, 18.33)	0.014
**Females, n = 2440**								
Normotensive-stable	Ref		Ref		Ref		Ref	
Prehypertension-stable	2.09 (1.18, 4.02)	0.017	2.07 (1.14, 4.05)	0.023	2.00 (1.10, 3.93)	0.032	1.91 (1.04, 3.76)	0.046
Stage I hypertension-increasing	3.36 (1.79, 6.68)	<0.001	2.90 (1.45, 6.10)	0.003	2.44 (1.18, 5.25)	0.019	2.21 (1.06, 4.78)	0.039
Stage II hypertension-increasing	6.85 (3.24, 14.78)	<0.001	4.95 (2.08, 11.91)	<0.001	3.63 (1.40, 9.42)	0.008	3.10 (1.19, 8.13)	0.021

Model 1: Unadjusted.

Model 2: Adjusted for age, gender (only for total), follow-up years, baseline SBP, and BMI.

Model 3: Adjusted for variables in Model 2 + smoking, alcohol drinking, physical-activity, energy intake, and antihypertensive drugs use.

Model 4: Adjusted for variables in Model 3 + BMI trajectory groups.

SBP, systolic blood pressure; BMI, body mass index.

**Table 3** shows the association between the joint-group of SBP and BMI trajectory membership and incident diabetes. We combined the two, consisting of the BMI overweight-increasing pattern and obesity-increasing pattern, into a group. Compared with the SBP normotensive-stable and BMI normal-stable pattern, individuals had a higher risk of diabetes in the stage I hypertension-increasing group (adjusted OR =2.29); the corresponding OR for this association in the stage II hypertension-increasing group was 3.76. When individuals had the BMI overweight or obesity increasing pattern, they had a significantly higher risk of diabetes in the stage I hypertension-increasing group (adjusted OR = 3.76) and the highest risk in the stage II hypertension-increasing group (adjusted OR = 6.56) compared with the SBP and BMI normal-stable pattern. When individuals had the BMI overweight or obesity increasing pattern, although within the prehypertension-stable SBP trajectory group, they still had the higher risk of diabetes (adjusted OR = 2.81) compared with the SBP and BMI normal-stable pattern.

**Table 3:** Odds Ratios and 95% CIs for Joint Trajectories of SBP and BMI Groups for Incident Diabetes.

		Model 1		Model 2		Model 3	
	% (n/N) *	OR (95%*CI*)	*P*	OR (95%*CI*)	*P*	OR (95%*CI*)	*P*
**BMI normal-stable**							
Normotensive-stable	1.8 (10/553)	Ref		Ref		Ref	
Prehypertension-stable	2.6 (34/1307)	1.45 (0.74, 3.12)	0.306	1.53 (0.77, 3.31)	0.250	1.49 (0.75, 3.24)	0.278
Stage I hypertension-increasing	3.9 (17/435)	2.21 (1.02, 5.05)	0.050	2.42 (1.08, 5.70)	0.035	2.29 (1.02, 5.42)	0.049
Stage II hypertension-increasing	8.3 (4/48)	4.94 (1.31, 15.43)	0.009	5.67 (1.46, 18.52)	0.006	5.30 (1.34, 17.71)	0.010
**BMI overweight/obesity increasing**							
Normotensive-stable	3.3 (7/209)	1.88 (0.68, 4.97)	0.206	1.39 (0.49, 3.73)	0.520	1.38 (0.49, 3.71)	0.530
Prehypertension-stable	6.6 (71/1074)	3.84 (2.06, 7.99)	<0.001	2.93 (1.50, 6.29)	0.003	2.81 (1.43, 6.06)	0.005
Stage I hypertension-increasing	8.9 (71/796)	5.32 (2.85, 11.06)	<0.001	4.09 (2.03, 9.02)	<0.001	3.76 (1.84, 8.39)	0.001
Stage II hypertension-increasing	16.7 (34/203)	10.92 (5.48, 23.76)	<0.001	7.75 (3.52, 18.26)	<0.001	6.56 (2.86, 15.99)	<0.001

Model 1: Unadjusted.

Model 2: Adjusted for age, gender, follow-up years, baseline SBP and BMI.

Model 3: Adjusted for variables in Model 2 +smoking, alcohol drinking, physical-activity, energy intake and antihypertensive drugs use.

SBP, systolic blood pressure; BMI, body mass index. *Diabetes event number divided by total number in each group.

**Table 4** presents the association of AUC quartiles of SBP growth curves and the risk of diabetes. Compared with the first quartile, the adjusted ORs were 1.66 and 1.82 of the third and fourth quartiles of the total SBP AUC, respectively, with adjustments to gender, age, smoking, alcohol drinking, physical activity, energy intake, antihypertensive drug use, and total BMI AUC. For the corresponding quantiles of the incremental SBP AUC, the adjusted ORs were 1.84 and 2.50 for the third and fourth quartiles, respectively, after additionally adjusting for baseline SBP and incremental BMI AUC.

**Table 4:** SBP AUC-odds ratios and 95% CIs for incident diabetes.

	Total AUC^a^		Baseline AUC^b^		Incremental AUC^c^	
	OR (95% CI)	*P*	OR (95% CI)	*P*	OR (95% CI)	*P*
Quartile 1	Ref		Ref		Ref	
Quartile 2	1.31 (0.82, 2.11)	0.266	0.99 (0.63, 1.56)	0.970	1.00 (0.61, 1.65)	0.998
Quartile 3	1.66 (1.06, 2.63)	0.029	1.33 (0.88, 2.05)	0.184	1.84 (1.19, 2.92)	0.008
Quartile 4	1.82 (1.15, 2.94)	0.012	1.46 (0.95, 2.28)	0.088	2.50 (1.61, 3.97)	<0.001

Quartile 1 group is the reference.

a Adjusted for age, gender, smoking, alcohol drinking, physical-activity, energy intake, antihypertensive drugs use, and total BMI AUC.

b Adjusted for age, gender, smoking, alcohol drinking, physical-activity, energy intake, antihypertensive drugs use, and baseline BMI.

c Adjusted for age, gender, smoking, alcohol drinking, physical-activity, energy intake, antihypertensive drugs use, incremental BMI AUC, and baseline SBP.

OR, odds ratio; CI, confidence interval; SBP, systolic blood pressure; BMI, body mass index.

**Figure 2:**
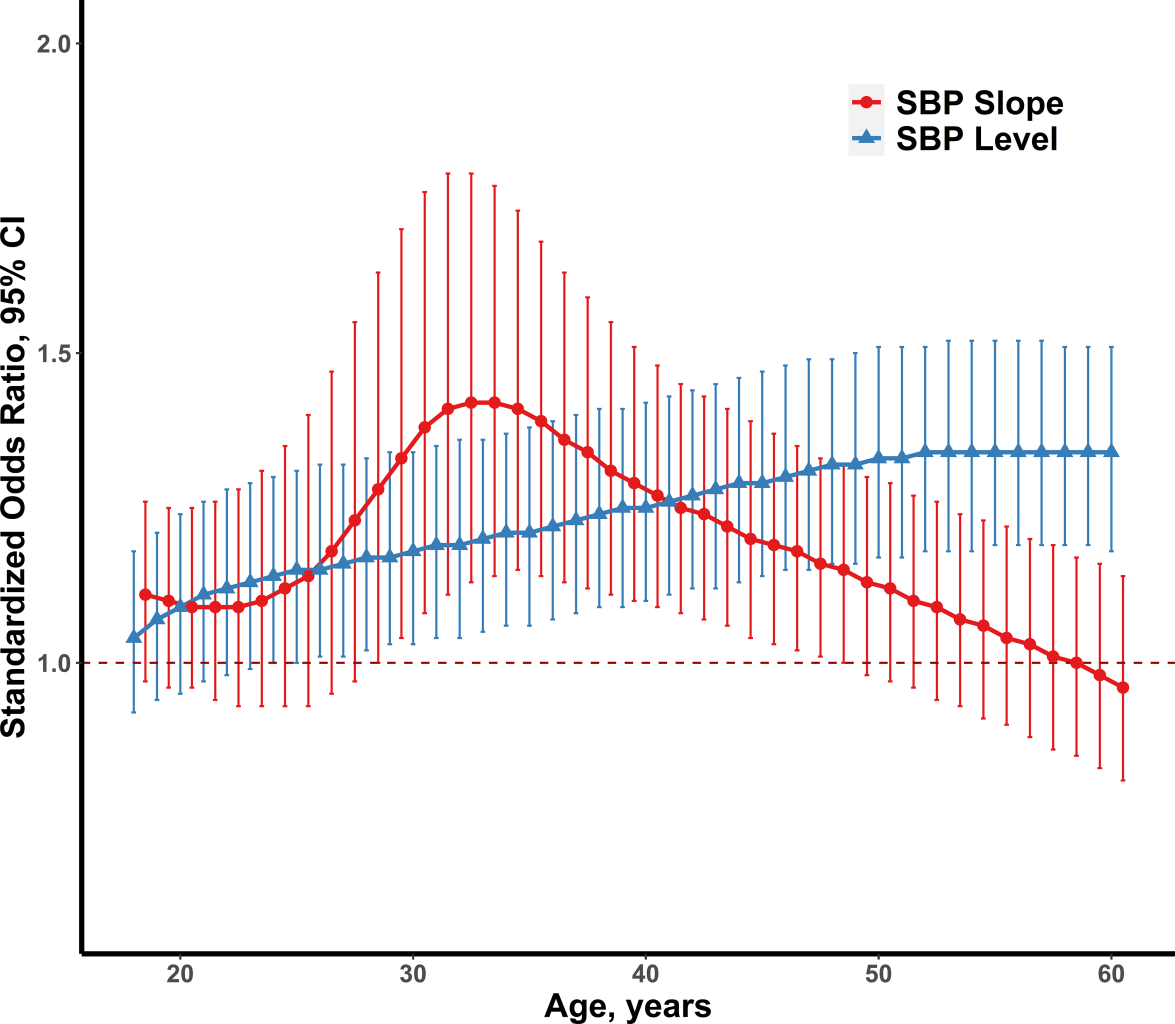
The standardized ORs and 95% CIs of model-estimated SBP levels and level-adjusted SBP slopes for adult diabetes (n=248), adjusting for gender, smoking, alcohol drinking, physical activity, energy intake, antihypertensive drugs use and baseline SBP (left), and model-estimated levels and linear slopes of BMI (right). SBP, systolic blood pressure; BMI, body mass index; OR, odds ratio; CI, confidence interval.

**Table S6** shows the model-estimated levels and linear slopes of SBP between normoglycemia and diabetes at the distinct age point separately. Compared with normoglycemia, diabetic individuals had the significant higher slopes of SBP during the follow-up and had the higher SBP levels except for those who were 18 years of age. **Figure 2** presents the standardized ORs (Cis) of model-estimated levels and level-adjusted linear slopes of SBP for incident diabetes. The standardized ORs of model-estimated SBP levels elevated gradually from 0.92 (1.04, 1.18) to 1.34 (1.18, 1.51), and this association between model-estimated SBP levels and diabetes became significantly positive at the age of 24 and above. The standardized ORs of model-estimated SBP slopes became significantly positive at the age of 28 (adjusted OR =1.28) and gradually increased to 1.42 (1.14, 1.77) at the age of 33, then declined to 1.15 (1.00, 1.32) at the age of 48.

The associations between SBP trajectory patterns and prediabetes were explored in our study as well. During the follow-up period, we identified 1,081 incident prediabetes cases. **Table S7** shows the follow-up characteristics of the study for incident prediabetes. Prediabetes had higher BMI and protein intake than normoglycemia.

Compared with the normotensive-stable SBP trajectory pattern, individuals had a higher risk of prediabetes (adjusted OR = 1.36) in the prehypertension-stable group (Model 4); the corresponding ORs in the stage I hypertension-increasing group and stage II hypertension-increasing group were 1.53 and 1.55, respectively (**Table S8**). When individuals had the BMI normal-stable pattern, they still had a higher risk of prediabetes in the SBP prehypertension-stable group (adjusted OR = 1.36) and an elevated risk in the stage II hypertension-increasing group (adjusted OR = 1.86) compared with the SBP normotensive-stable group (**Table S9**). Compared with the SBP and BMI normal-stable pattern, individuals were at significantly higher risks of prediabetes in the prehypertension-stable group (adjusted OR = 1.94), the stage I hypertension-increasing group (adjusted OR = 2.03), and the stage II hypertension-increasing group (adjusted OR =2. 38) who had the BMI overweight or obesity increasing pattern.

## Discussion

In this community-based longitudinal analysis, we identified four distinct trajectories of SBP over 18–60 years of age, which were significantly associated with incident diabetes in later life, independent of baseline SBP and BMI levels. Compared with subjects who remained at SBP <120 mmHg consistently in the normotensive-stable group, individuals within the stage II hypertension-increasing trajectory group had the highest risk of diabetes: the risk was 3.91-fold higher. Moreover, individuals in the prehypertension-stable trajectory exhibited a normal SBP range (<140 mmHg); they still had a significantly higher risk of diabetes (adjusted OR = 1.82) compared with the normotensive-stable group. Specifically, participants with a dynamic, rapid increase in SBP demonstrated higher glucose by late life compared with those with stable SBP. Systolic BP trajectories predict incident diabetes better than SBP at a single time point and single BMI trajectory. We also explored the relationship of SBP trajectories with prediabetes and generated similarly significant results.

Hypertension and diabetes share common risk factors including obesity, BP, and blood lipids, and they often coexist (14). The relationship between BP as well as BP progression and new-onset diabetes has been evaluated in previous studies (12–15). To the best of our knowledge, prehypertension and hypertension are important risk factors in developing diabetes, independent of baseline glucose status, gender, and BMI (12–15). The development of diabetes risk was significantly greater in the poorly controlled group than the well-controlled group (14). There was an additive interaction between BMI and hypertensive disorders of pregnancy for the development of type 2 diabetes in women with obesity (40). Sequentially, in addition to hypertension, BP and BP progression are also important predictors of new-onset diabetes. Moreover, the relationship is independent of BMI and the other metabolic syndrome components (12–15). These findings suggest that aggressive BP control, especially as early as possible, is necessary to prevent the development of diabetes. However, no previous studies have investigated the association between longitudinal profiles of SBP and incident diabetes.

In the current study, distinct longitudinal SBP patterns were identified, and the gradient of diabetes risk was observed across the four trajectory groups. The SBP trajectory patterns were similar in the previous analyses (27–30). Li et al. explored the association between long-term SBP trajectory patterns and the risk of intracerebral hemorrhage based on the Kailuan Study, which was a community-based longitudinal cohort study, in Tangshan City, China (27). Tielemans et al. identified four distinct SBP trajectories and examined their association with cardiovascular mortality and life-years lost based on the Minnesota Business and Professional Men Study and the Zutphen Study (29), and the trajectory growth patterns were similar to our study. Four unique long-term SBP trajectories were found, and they are significant predictors of CVD and all-cause mortality (the Rancho Bernardo Study, USA) (41). In the Framingham Heart Study, four SBP trajectory groups were identified (42), and SBP growth trends were similar to those of the current study, but the associations between trajectories and incident diabetes were not investigated.

Trajectories of SBP reflect the dynamic trend of SBP over time during the life course, taking into consideration multiple aspects of life course patterns such as baseline levels and slopes. In the current study, compared with the normotensive-stable group, individuals who kept SBP <140 mmHg throughout still had an elevated diabetes risk in the prehypertension-stable group. Individuals with the stage I hypertension-increasing SBP trajectory had a lower diabetes risk compared with subjects in the stage II hypertension-increasing group, although they had a higher baseline SBP. The results demonstrate that using a single SBP measurement to predict diabetes risk may lead to the misclassification of risk groups, and the long-term SBP changes provide more insight into the evolution of risk because trajectories capture both the levels and longitudinal changes of SBP.

The relationship of the joint group of SBP and BMI trajectories with incident diabetes was also explored in our study, and three distinct trajectories of BMI were identified, which were similar with previous analyses (18, 43). The associations between SBP trajectories and diabetes were robust and remained significant. We found that individuals within the same SBP trajectory pattern had distinct risks of developing diabetes because of different BMI trajectory groups. People within the stage II hypertension-increasing SBP trajectory group had the highest risk of diabetes (adjusted OR =7.75, p < 0.001); they had the BMI overweight/obesity increasing trajectory; however, the correspondingly adjusted OR was 5.67 in the BMI normal-stable group. These results indicate that joint trajectories of SBP and BMI have additional value for predicting diabetes compared with a single SBP trajectory pattern and demonstrate the importance of controlling blood pressure as well as weight.

Previous research studies have demonstrated the importance of long-term exposure to SBP on incident CVD (44–46). Using long-term cumulative SBP exposure, rather than a single measurement, can modestly improve the ability of CVD risk prediction models. Since the SBP level rises with age for most individuals, the association of long-term SBP exposure throughout the life course with diabetes is also of particular interest. To define the exposure to SBP, the cumulative total and incremental SBP AUC were calculated. Total AUC is an assessment of long-term burden, and incremental AUC (total AUC-baseline AUC) combines linear and non-linear long-term trends over time (47). Compared with the first quartile, the adjusted ORs were 1.84 and 2.50 for the third and fourth quartiles of long-term burden (incremental AUC) of SBP, which were significantly related to incident diabetes. The adjusted significant ORs of the third and fourth quartiles of the SBP total AUC were 1.66 and 1.82, respectively. These findings indicate that the cumulative SBP load plays a major role in determining the incident diabetes risk, and the risk of future incident diabetes at a given age increases with the accumulated area under the SBP versus age curve. Importantly, the long-term burden of SBP cannot be neglected, and we should attach importance to early BP management and active control to prevent incident diabetes.

Several pathophysiological mechanisms may underlie the association between BP and impaired glucose metabolism (48), although the direct causal link has not been established. New-onset diabetes is associated with the markers of endothelial dysfunction, and BP and hypertension are closely related to endothelial dysfunction (49). Changes in endothelial permeability and diminished peripheral blood flow caused by hypertension may limit insulin release and promote insulin resistance in metabolically active tissues (50). In addition, oxidative stress associated with hypertension is postulated to play a key role in pancreatic b-cell dysfunction (51). Thus, there could be potential links between BP and diabetes.

In most previous studies, no attention was paid to the association of SBP levels and slopes at distinct ages for predicting diabetes. This study provided a unique perspective on the relationship between blood pressure and diabetes in early adulthood and explored the effect of the level and linear slope of BP at different points of adult age (18–60 years) on predicting diabetes risk. As the model-estimated SBP levels increased gradually, the independent effects of SBP slopes decreased in developing later-life diabetes. The SBP slope indicates that the velocity of SBP levels changes over time. By analyzing level-adjusted SBP slopes, the current study captured the critical period during 30–35 years of age where the association between the SBP slope and incident diabetes in this period was significant and had a greater OR than the SBP level. This highlights the importance of considering SBP slopes when assessing diabetes risk in young adults.

Increased adiposity is the strongest risk factor for developing diabetes (52). However, baseline BP and BP progression are significant predictors of new-onset diabetes among initially healthy individuals (13–15). Therefore, it is necessary to take SBP into consideration when identifying individuals at high risk for diabetes, and the corresponding control and management. To the best of our knowledge, the current study was the first to explore the longitudinal trajectories of SBP in young adulthood and its effect on diabetes. The study provides new insights in developing diabetes in early adulthood and emphasizes the significance of the level-independent SBP trajectories during 18–60 years of age for assessing diabetes risk. Maintaining a high SBP level and/or increasing SBP rapidly may increase the risk of diabetes. Consequently, in addition to emphasizing the importance of weight control, interventions targeting blood pressure are of significance for individuals at high risk for diabetes.

### Strengths and limitations

The current study has important strengths including the large study sample size and long follow-up years. Participants have been examined at least five times, and distinct trajectory subgroups can be identified by the GBTM to explore the relationship between the trajectory trends and health outcomes. Previous studies almost focused on the relationship of BMI growth trajectories or cross-sectional blood pressure with clinical outcomes. In our study, we emphasized the growth patterns of blood pressure. We also considered the significance of linear slopes and levels of SBP at each age point between 18 and 60 years for predicting incident diabetes. However, the study has certain limitations. Firstly, this study was only investigated in Chinese participants, which limited the generalizability to other ethnic population. Secondly, incident diabetes was partly defined according to self-report, and HbA1c and glucose biomarkers, which were only measured in the 2009 survey, were used to identify diabetes. However, the incidence of new-onset diabetes in our follow-up was only 5.4%, which was understandable because we excluded the individuals with diabetes at baseline and included participants with more than four follow-up visits who developed no diabetic outcomes during the follow-up period of less than four visits. Moreover, the prevalence of diabetes was 0.67% in 1980 in the Chinese population (8). In subsequent national surveys conducted in 1994, 2000–2001, 2007, and 2013, the prevalence of diabetes was reported to be 2.5%, 5.5%, 9.7%, and 10.9% (3, 53–55), respectively. The latest published nationwide estimation was 12.4% in 2018 (9). Therefore, we expected that the low incidence of diabetes cases may not affect the results of this study. Thirdly, like any observational study, it was limited by the possibility of residual confounding and measurement error.

## Conclusions

Although BP has been a well-known risk factor for diabetes, the current study suggests that an individual’s longitudinal SBP trajectory may provide additional information about his or her risk of development of diabetes. We identified four distinct trajectories of SBP during young adulthood in a Chinese population, and the high-risk diabetes group was composed of participants who had the higher growth slopes and higher levels of blood pressure. In particular, long-term exposure to BP levels in the prehypertension range or higher was strongly associated with diabetes. The findings demonstrate that long-term SBP trajectory is a significant predictor for incident diabetes, and highlight the significant importance of paying more attention to changing slopes of SBP and maintaining optimal blood pressure levels during young adulthood for preventing diabetes. Additional research is needed to explore the effect of healthy diet and lifestyle modification, and timing of intervention on lifetime trajectories in BP and outcomes.

## Data availability statement

The datasets presented in this study can be found in online repositories. The names of the repository/repositories and accession number(s) can be found below: Data are public and the data sets analyzed in the current study are available in the web: https://www.cpc.unc.edu/projects/china.

## Ethics statement

The studies involving human participants were reviewed and approved by The protocol was approved by the Ethics Committee of the National Institute for Nutrition and Health, China CDC (Number 201524). The patients/participants provided their written informed consent to participate in this study.

## Author contributions

YL, CL and TZ generated the hypothesis, directed implementation, and wrote the manuscript. YL, CL, BF, JL and YQ contributed to analytic strategy and statistical analyses. YL, CL, BF, JL, YQ XC YC and TZ supervised the field activities and data collection and edited the manuscript. All authors contributed to the article and approved the submitted version.

## Funding

This study was supported by grants 82222064 and 81973147 from the National Natural Science Foundation of China and the Cheeloo Young Scholars Program of Shandong University.

## Acknowledgments

The China Health and Nutrition Survey is a joint effort of many investigators and staff members whose contributions are acknowledged. We appreciate the participants and their families in the CHNS for the provision of data.

## Conflict of interest

The authors declare that the research was conducted in the absence of any commercial or financial relationships that could be construed as a potential conflict of interest.

## Publisher’s note

All claims expressed in this article are solely those of the authors and do not necessarily represent those of their affiliated organizations, or those of the publisher, the editors and the reviewers. Any product that may be evaluated in this article, or claim that may be made by its manufacturer, is not guaranteed or endorsed by the publisher.

## Abbreviations

DM, diabetes mellitus; HbA1c, hemoglobin a1c; BP, blood pressure; SBP, systolic blood pressure; DBP, diastolic blood pressure; BMI, body mass index; OR, odds ratio; CI, confidence interval; AUC, area under the curve.
