# Supplementary material for: Life-course blood pressure trajectories and incident diabetes: A longitudinal cohort in a Chinese population
Source: Front Endocrinol (Lausanne). 2022 Nov 11;13:1035890. doi: 10.3389/fendo.2022.1035890 (PMC9691649; doi:10.3389/fendo.2022.1035890)
Supplement: Supplementary file 1 [file DataSheet_1.docx]

***Supplementary Material***

4937 people included in BMI trajectory analysis

The China and Nutrition Survey (n = 33348)

11173 people excluded:

<18 years or >60 years

858 people excluded:

missing information in BMI

16380 people excluded: prevalent diabetes

at baseline and < 5 follow-up visits

990 people excluded:

missing information in SBP

16504 people excluded: prevalent diabetes

at baseline and < 5 follow-up visits

4681 people included in SBP trajectory analysis

Take the intersection: 4625 people included

in the analysis

**Figure S1.** Flow chart of the study population selection.


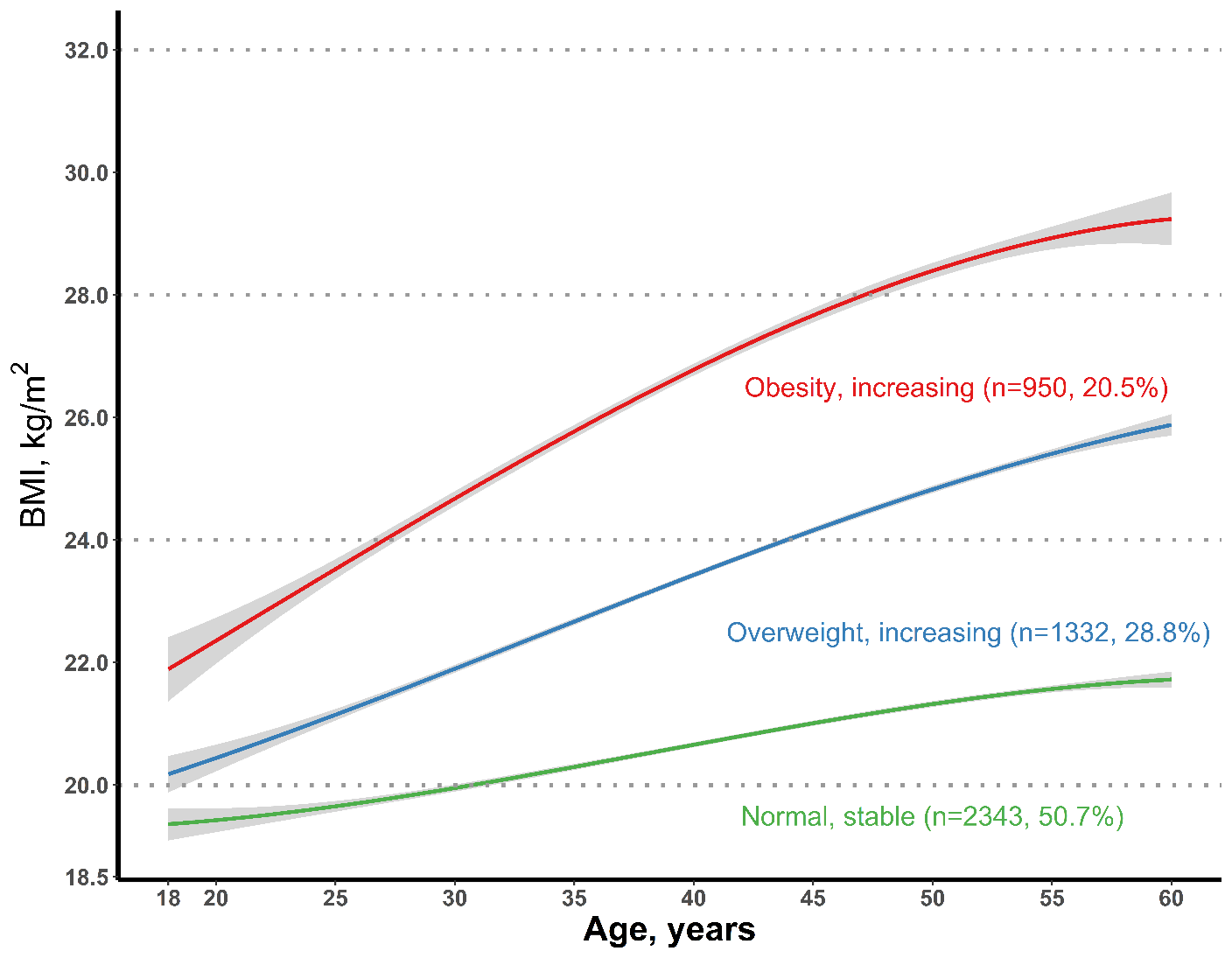


**Figure S2.** Predicted trajectories of BMI during young adulthood. BMI indicates body mass index.

**Table S1.** Group-based trajectory models (GBTM) results of SBP model fitting process.

| Number of groups | Trajectory shapes^1^ | Log-Lik | BIC^2^ (N=4625) | BIC^3^ (N=30083) | %Participants per class | Mean posterior probabilities |
| --- | --- | --- | --- | --- | --- | --- |
| 1 | 1 | -121838.00 | -121850.66 | -121853.47 | 100 | 1 |
| 1 | 2 | -121738.76 | -121755.64 | -121759.39 | 100 | 1 |
| 1 | 3 | -121728.10 | -121749.20 | -121753.88 | 100 | 1 |
| 2 | 1 1 | -119098.14 | -119123.46 | -119129.08 | 67.37/32.63 | 0.94/0.89 |
| 2 | 2 2 | -118917.18 | -118950.93 | -118958.42 | 66.16/33.84 | 0.94/0.89 |
| 2 | 2 3 | -118907.37 | -118945.35 | -118953.77 | 66.14/33.86 | 0.94/0.89 |
| 3 | 1 1 1 | -118298.73 | -118336.71 | -118345.13 | 38.79/51.31/9.90 | 0.87/0.85/0.87 |
| 3 | 2 2 2 | -118098.96 | -118149.60 | -118160.83 | 39.52/50.66/9.82 | 0.87/0.85/0.87 |
| 3 | 2 2 3 | -118078.76 | -118133.61 | -118145.78 | 39.81/50.53/9.67 | 0.87/0.85/0.87 |
| 4 | 1 1 1 1 | -118055.87 | -118106.5 | -118117.74 | 16.80/52.02/26.00/5.19 | 0.82/0.80/0.78/0.87 |
| **4** | **1 2 2 2** | **-117819.15** | **-117882.44** | **-117896.48** | **16.48/26.62/51.48/5.43** | **0.83/0.78/0.80/0.87** |
| 4 | 3 3 3 3† | -117795.83 | -117880.22 | -117898.95 |  |  |
| 5 | 1 1 1 1 1 | -117979.37 | -118042.67 | -118056.71 | 52.95/25.06/17.32/4.37/0.30 | 0.80/0.79/0.83/0.86/0.89 |
| 5 | 2 2 1 1 1 | -117683.06 | -117754.79 | -117770.71 | 52.17/6.66/17.12/19.78/4.26 | 0.75/0.74/0.82/0.72/0.87 |
| 5 | 1 2 3 2 3 | -117610.78 | -117699.39 | -117719.05 | 15.98/50.72/3.33/26.90/3.07 | 0.82/0.80/0.81/0.77/0.83 |
| 6 | 0 0 0 0 0 0* | -120452.57 | -120503.20 | -120514.44 |  |  |

^1^Trajectory shapes: 0 = zero-order; 1 = linear; 2 = quadratic.

^2^BIC = Bayesian information criterion (for the total number of participants).

^3^BIC = Bayesian information criterion (for the total number of observations).

*One or more of the groups had a very small proportion of the participants.

†The P value of this order term is not significant.

The best fitting model is highlighted in bold characters.

**Table S2.** Parameter estimates for the best fitting 4-class group-based trajectory model of SBP.

| Trajectory group | Polynomial degree | Estimate | Standard Error | T for H0: parameter=0 | *P* |
| --- | --- | --- | --- | --- | --- |
| 1 | Intercept | 91.714 | 0.944 | 97.161 | <0.001 |
|  | Linear | 0.307 | 0.021 | 14.303 | <0.001 |
| 2 | Intercept | 133.538 | 3.300 | 40.471 | <0.001 |
|  | Linear | -1.408 | 0.162 | -8.683 | <0.001 |
|  | Quadratic | 0.027 | 0.002 | 13.933 | <0.001 |
| 3 | Intercept | 110.465 | 2.201 | 50.199 | <0.001 |
|  | Linear | -0.419 | 0.106 | -3.948 | <0.001 |
|  | Quadratic | 0.012 | 0.001 | 9.137 | <0.001 |
| 4 | Intercept | 97.420 | 8.220 | 11.851 | <0.001 |
|  | Linear | 0.480 | 0.408 | 1.176 | 0.240 |
|  | Quadratic | 0.012 | 0.005 | 2.409 | 0.016 |

**Table S3.** Baseline characteristics of participants included and excluded.

| Variable | Included | Excluded | *P* |
| --- | --- | --- | --- |
| n | 4625 | 28723 |  |
| Age, y | 32.6 (7.2) | 30.9 (22.5) | <0.001 |
| Males, n (%) | 2185 (47.2) | 13809 (48.1) | 0.300 |
| BMI, kg/m^2^ | 21.7 (2.5) | 20.9 (4.7) | <0.001 |
| SBP, mm Hg | 111.7 (13.2) | 115.6 (19.6) | <0.001 |
| DBP, mm Hg | 73.1 (9.8) | 74.6 (12.0) | <0.001 |
| Smoker, n (%) | 537 (11.6) | 5048 (17.6) | <0.001 |
| Drinker, n (%) | 602 (13.0) | 5820 (20.3) | <0.001 |
| Hypertension, n (%) | 152 (3.3) | 3182 (13.9) | <0.001 |

Data are means ± SD, or n (%).

BMI, body mass index; SBP, systolic blood pressure; DBP, diastolic blood pressure.

**Table S4.** Descriptive data of baseline and follow-up characteristics by incident diabetes at follow-up.

| Variable | Total  (n=4625) | Normal  (n=4377) | Diabetes  (n=248) | *P* |
| --- | --- | --- | --- | --- |
| **Baseline** |  |  |  |  |
| Age, y | 32.6 (7.2) | 32.61 (7.3) | 33.3 (6.2) | 0.162 |
| BMI, kg/m^2^ | 21.7 (2.5) | 21.6 (2.5) | 23.2 (2.9) | <0.001 |
| SBP, mm Hg | 111.7 (13.2) | 111.5 (13.1) | 114.8 (14.1) | <0.001 |
| DBP, mm Hg | 73.1 (9.8) | 73.0 (9.7) | 75.4 (9.9) | <0.001 |
| Smoker, n (%) | 537 (11.6) | 515 (11.8) | 22 (8.9) | 0.200 |
| Drinker, n (%) | 602 (13.0) | 571 (13.0) | 31 (12.5) | 0.880 |
| Hypertension, n (%) | 152 (3.3) | 138 (3.2) | 14 (5.6) | 0.050 |
| **Follow-up** |  |  |  |  |
| Age, y | 50.3 (7.3) | 50.2 (7.4) | 51.2 (6.3) | 0.039 |
| BMI, kg/m^2^ | 23.8 (3.9) | 23.7 (3.9) | 25.6 (3.6) | <0.001 |
| SBP, mm Hg | 123.0 (17.5) | 122.5 (17.3) | 131.7 (17.9) | <0.001 |
| DBP, mm Hg | 80.4 (11.2) | 80.1 (11.1) | 85.0 (12.6) | <0.001 |
| Smoker, n (%) | 1500 (32.4) | 1418 (32.4) | 82 (33.1) | 0.882 |
| Drinker, n (%) | 1668 (36.1) | 1571 (35.9) | 97 (39.1) | 0.337 |
| Hypertension, n (%) | 1028 (22.2) | 918 (21.0) | 110 (44.4) | <0.001 |
| FPG, mmol/L | 5.8 (1.9) | 5.1 (0.6) | 7.7 (2.7) | <0.001 |
| HbA1c, % | 5.9 (1.5) | 5.5 (0.4) | 7.1 (2.3) | <0.001 |
| Follow up, y | 17.8 (3.8) | 17.7 (3.8) | 18.1 (3.0) | 0.197 |

Data are means ± SD, or n (%).

BMI, body mass index; SBP, systolic blood pressure; DBP, diastolic blood pressure; FPG, fasting plasma glucose, Hb1Ac, Hemoglobin A1c.

**Table S5.** Characteristics of participants by the latent SBP pattern classes.

| Variable | Normotensive  -stable | Prehypertension  -stable | Stage I hypertension-increasing | Stage II hypertension-increasing | *P* |
| --- | --- | --- | --- | --- | --- |
| n | 762 | 2381 | 1231 | 251 |  |
| Age, ys | 34.7 (6.8) | 32.8 (7.2) | 31.35 (7.4) | 31.6 (6.9) | <0.001 |
| Males, n (%) | 194 (25.5) | 1098 (46.1) | 765 (62.1) | 128 (51.0) | <0.001 |
| BMI, kg/m^2^ | 20.82 (2.1) | 21.51 (2.4) | 22.23 (2.7) | 23.33 (2.8) | <0.001 |
| SBP, mm Hg | 101.47 (10.3) | 109.90 (10.6) | 118.65 (12.2) | 125.0 (18.3) | <0.001 |
| DBP, mm Hg | 67.2 (7.9) | 72.0 (8.6) | 76.9 (9.4) | 82.1 (12.6) | <0.001 |
| Smoker, n (%) | 29 (3.8) | 250 (10.5) | 220 (17.9) | 38 (15.1) | <0.001 |
| Drinker, n (%) | 53 (7.0) | 268 (11.3) | 235 (19.1) | 46 (18.3) | <0.001 |
| Hypertension, n (%) | 2 (0.3) | 30 (1.3) | 72 (5.8) | 48 (19.1) | <0.001 |
| Physical-activity | 199 (26.1) | 685 (28.8) | 308 (25.0) | 68 (27.1) | <0.001 |
| Light, n (%) | 173 (22.7) | 733 (30.8) | 424 (34.4) | 88 (35.1) |  |
| Moderate, n (%) | 390 (51.2) | 963 (40.4) | 499 (40.5) | 95 (37.8) |  |
| Heavy, n (%) | 199 (26.1) | 685 (28.8) | 308 (25.0) | 68 (27.1) |  |
| Energy intake, kcal/d | 2508.4 (525.0) | 2516.6 (488.0) | 2550.3 (534.3) | 2514.9 (363.5) | 0.193 |
| Carbohydrate intake, g/d | 400.9 (66.4) | 404.1 (85.8) | 415.4 (93.7) | 407.6 (72.8) | <0.001 |
| Fat intake, g/d | 65.6 (49.7) | 63.7 (22.8) | 612.0 (23.6) | 61.41 (20.1) | 0.036 |
| Hypertensive, n (%) | 74.5 (11.3) | 76.5 (32.2) | 77.6 (40.8) | 76.0 (15.2) | 0.201 |
| FPG*, mmol/L | 5.2 (1.2) | 5.7 (1.87) | 6.1 (2.0) | 7.1 (2.6) | <0.001 |
| HbA1c*, % | 5.7 (0.9) | 5.9 (1.7) | 6.1 (1.3) | 6.5 (1.6) | 0.003 |
| Diabetes, n (%) | 17 (2.2) | 105 (4.4) | 88 (7.1) | 38 (15.1) | <0.001 |
| Follow up, y | 18.0 (3.7) | 17.7 (3.8) | 17.6 (3.8) | 18.1 (3.7) | 0.034 |

Data are means ± SD, or n (%).

* Follow-up information.

BMI, body mass index; SBP, systolic blood pressure; DBP, diastolic blood pressure; FPG, fasting plasma glucose; Hb1Ac, Hemoglobin A1c.

**Table S6.** Model-estimated levels and linear slopes of SBP in means (SD) by incident diabetes at follow-up.

|  | SBP Level (mm Hg) | | | SBP Slope (mm Hg/yr) | | |
| --- | --- | --- | --- | --- | --- | --- |
| Age (yr) | Normoglycemia | Diabetes | *P* | Normoglycemia | Diabetes | *P* |
| 18 | 108.59 (2.32) | 108.82 (2.10) | 0.131 | -0.07 (0.55) | 0.11 (0.62) | <0.001 |
| 19 | 108.54 (2.31) | 108.94 (2.15) | 0.008 | -0.04 (0.53) | 0.13 (0.59) | <0.001 |
| 20 | 108.52 (2.42) | 109.09 (2.35) | <0.001 | -0.01 (0.51) | 0.16 (0.57) | <0.001 |
| 21 | 108.53 (2.62) | 109.27 (2.64) | <0.001 | 0.02 (0.49) | 0.19 (0.54) | <0.001 |
| 22 | 108.57 (2.88) | 109.47 (3.00) | <0.001 | 0.05 (0.47) | 0.22 (0.52) | <0.001 |
| 23 | 108.63 (3.18) | 109.71 (3.38) | <0.001 | 0.08 (0.45) | 0.25 (0.50) | <0.001 |
| 24 | 108.73 (3.49) | 109.97 (3.77) | <0.001 | 0.11 (0.43) | 0.28 (0.48) | <0.001 |
| 25 | 108.86 (3.81) | 110.26 (4.17) | <0.001 | 0.14 (0.41) | 0.31 (0.46) | <0.001 |
| 26 | 109.02 (4.14) | 110.59 (4.56) | <0.001 | 0.17 (0.39) | 0.34 (0.44) | <0.001 |
| 27 | 109.20 (4.46) | 110.94 (4.93) | <0.001 | 0.20 (0.37) | 0.37 (0.42) | <0.001 |
| 28 | 109.42 (4.77) | 111.32 (5.30) | <0.001 | 0.23 (0.36) | 0.39 (0.40) | <0.001 |
| 29 | 109.66 (5.07) | 111.73 (5.65) | <0.001 | 0.26 (0.34) | 0.42 (0.38) | <0.001 |
| 30 | 109.94 (5.36) | 112.16 (5.99) | <0.001 | 0.29 (0.33) | 0.45 (0.37) | <0.001 |
| 31 | 110.25 (5.63) | 112.63 (6.31) | <0.001 | 0.32 (0.32) | 0.48 (0.36) | <0.001 |
| 32 | 110.58 (5.90) | 113.13 (6.61) | <0.001 | 0.35 (0.31) | 0.51 (0.34) | <0.001 |
| 33 | 110.94 (6.15) | 113.65 (6.90) | <0.001 | 0.38 (0.30) | 0.54 (0.34) | <0.001 |
| 34 | 111.34 (6.40) | 114.20 (7.18) | <0.001 | 0.41 (0.30) | 0.57 (0.33) | <0.001 |
| 35 | 111.76 (6.63) | 114.78 (7.44) | <0.001 | 0.44 (0.29) | 0.60 (0.32) | <0.001 |
| 36 | 112.21 (6.85) | 115.40 (7.70) | <0.001 | 0.47 (0.29) | 0.63 (0.32) | <0.001 |
| 37 | 112.70 (7.06) | 116.04 (7.94) | <0.001 | 0.50 (0.30) | 0.65 (0.32) | <0.001 |
| 38 | 113.21 (7.27) | 116.70 (8.16) | <0.001 | 0.53 (0.30) | 0.68 (0.33) | <0.001 |
| 39 | 113.75 (7.46) | 117.40 (8.38) | <0.001 | 0.56 (0.31) | 0.71 (0.33) | <0.001 |
| 40 | 114.32 (7.65) | 118.13 (8.59) | <0.001 | 0.59 (0.31) | 0.74 (0.34) | <0.001 |
| 41 | 114.93 (7.84) | 118.88 (8.80) | <0.001 | 0.62 (0.33) | 0.77 (0.35) | <0.001 |
| 42 | 115.56 (8.03) | 119.67 (9.00) | <0.001 | 0.65 (0.34) | 0.80 (0.36) | <0.001 |
| 43 | 116.22 (8.21) | 120.48 (9.20) | <0.001 | 0.68 (0.35) | 0.83 (0.38) | <0.001 |
| 44 | 116.91 (8.39) | 121.32 (9.39) | <0.001 | 0.71 (0.37) | 0.86 (0.39) | <0.001 |
| 45 | 117.63 (8.58) | 122.19 (9.59) | <0.001 | 0.73 (0.38) | 0.89 (0.41) | <0.001 |
| 46 | 118.38 (8.77) | 123.09 (9.79) | <0.001 | 0.76 (0.40) | 0.91 (0.43) | <0.001 |
| 47 | 119.16 (8.97) | 124.02 (10.00) | <0.001 | 0.79 (0.42) | 0.94 (0.45) | <0.001 |
| 48 | 119.97 (9.18) | 124.98 (10.21) | <0.001 | 0.82 (0.44) | 0.97 (0.47) | <0.001 |
| 49 | 120.80 (9.39) | 125.97 (10.43) | <0.001 | 0.85 (0.46) | 1.00 (0.49) | <0.001 |
| 50 | 121.67 (9.62) | 126.98 (10.67) | <0.001 | 0.88 (0.48) | 1.03 (0.51) | <0.001 |
| 51 | 122.57 (9.87) | 128.03 (10.92) | <0.001 | 0.91 (0.50) | 1.06 (0.54) | <0.001 |
| 52 | 123.50 (10.13) | 129.10 (11.18) | <0.001 | 0.94 (0.52) | 1.09 (0.56) | <0.001 |
| 53 | 124.45 (10.41) | 130.20 (11.47) | <0.001 | 0.97 (0.55) | 1.12 (0.58) | <0.001 |
| 54 | 125.44 (10.71) | 131.33 (11.77) | <0.001 | 1.00 (0.57) | 1.15 (0.61) | <0.001 |
| 55 | 126.46 (11.04) | 132.49 (12.10) | <0.001 | 1.03 (0.59) | 1.17 (0.63) | <0.001 |
| 56 | 127.50 (11.39) | 133.68 (12.45) | <0.001 | 1.06 (0.61) | 1.20 (0.66) | <0.001 |
| 57 | 128.58 (11.76) | 134.90 (12.83) | <0.001 | 1.09 (0.64) | 1.23 (0.68) | 0.001 |
| 58 | 129.68 (12.16) | 136.15 (13.24) | <0.001 | 1.12 (0.66) | 1.26 (0.71) | 0.001 |
| 59 | 130.82 (12.59) | 137.42 (13.68) | <0.001 | 1.15 (0.68) | 1.29 (0.74) | 0.002 |
| 60 | 131.98 (13.04) | 138.73 (14.15) | <0.001 | 1.18 (0.71) | 1.32 (0.76) | 0.003 |

SBP, systolic blood pressure.

**Table S7.** Baseline characteristics by incident diabetes and prediabetes at follow-up.

| Variable | Total | Normoglycemia | Prediabetes | *P* |
| --- | --- | --- | --- | --- |
| n | 4377 | 3296 | 1081 |  |
| Age, ys | 32.6 (7.3) | 32.8 (7.5) | 32.0 (6.5) | 0.002 |
| Males, n (%) | 2061 (47.1) | 1544 (46.8) | 517 (47.8) | 0.599 |
| BMI, kg/m^2^ | 21.6 (2.5) | 21.54 (2.4) | 21.79 (2.5) | 0.004 |
| SBP, mm Hg | 111.5 (13.1) | 111.34 (13.2) | 111.90 (12.9) | 0.228 |
| DBP, mm Hg | 73.0 (9.7) | 72.91 (9.7) | 73.09 (9.7) | 0.596 |
| Smoker, n (%) | 515 (11.8) | 384 (11.7) | 131 (12.1) | 0.719 |
| Drinker, n (%) | 571 (13.0) | 427 (13.0) | 144 (13.3) | 0.796 |
| Hypertension, n (%) | 138 (3.2) | 113 (3.4) | 25 (2.3) | 0.085 |
| Physical-activity |  |  |  | 0.001 |
| Light, n (%) | 1192 (27.2) | 943 (28.6) | 249 (23.0) |  |
| Moderate, n (%) | 1325 (30.3) | 973 (29.5) | 352 (32.6) |  |
| Heavy, n (%) | 1860 (42.5) | 1380 (41.9) | 480 (44.4) |  |
| Energy intake, kcal/d | 2520.8 (507.5) | 2514.8 (492.5) | 2539.0 (550.5) | 0.174 |
| Carbohydrate intake, g/d | 406.1 (85.0) | 405.8 (82.8) | 406.9 (91.3) | 0.701 |
| Fat intake, g/d | 63.4 (30.0) | 63.2 (31.8) | 64.06 (21.9) | 0.397 |
| Protein intake, g/d | 76.3 (32.5) | 75.6 (26.9) | 78.52 (45.5) | 0.011 |
| FPG*, mmol/L | 5.1 (0.6) | 4.83 (0.5) | 5.34 (0.7) | <0.001 |
| HbA1c*, % | 5.5 (0.4) | 5.24 (0.4) | 5.77 (0.4) | <0.001 |
| Follow up, ys | 17.7 (3.8) | 17.21 (3.7) | 19.36 (3.6) | <0.001 |

Data are means ± SD, or n (%).

* Follow-up information.

BMI, body mass index; SBP, systolic blood pressure; DBP, diastolic blood pressure; FPG, fasting plasma glucose; Hb1Ac, Hemoglobin A1c.

**Table S8.** Odds ratios and 95% CIs for trajectories of SBP groups for incident prediabetes.

|  | Model 1 |  | Model 2 |  | Model 3 |  | Model 4 |  |
| --- | --- | --- | --- | --- | --- | --- | --- | --- |
|  | OR (95%*CI*) | *P* | OR (95%*CI*) | *P* | OR (95%*CI*) | *P* | OR (95%*CI*) | *P* |
| **Total, n = 3296** |  |  |  |  |  |  |  |  |
| Normotensive-stable | Ref |  | Ref |  | Ref |  | Ref |  |
| Prehypertension-stable | 1.32 (1.07, 1.62) | 0.008 | 1.40 (1.12, 1.75) | 0.003 | 1.40 (1.13, 1.76) | 0.003 | 1.36 (1.09, 1.71) | 0.007 |
| Stage I hypertension-increasing | 1.58 (1.27, 1.98) | <0.001 | 1.72 (1.32, 2.24) | <0.001 | 1.68 (1.29, 2.21) | <0.001 | 1.53 (1.16, 2.02) | 0.002 |
| Stage II hypertension-increasing | 2.03 (1.44, 2.84) | <0.001 | 1.94 (1.32, 2.86) | 0.001 | 1.86 (1.22, 2.82) | 0.003 | 1.55 (1.01, 2.36) | 0.044 |
| **Males, n = 2061** |  |  |  |  |  |  |  |  |
| Normotensive-stable | Ref |  | Ref |  | Ref |  | Ref |  |
| Prehypertension-stable | 1.40 (0.95, 2.11) | 0.098 | 1.39 (0.92, 2.14) | 0.126 | 1.33 (0.88, 2.06) | 0.182 | 1.33 (0.88, 2.06) | 0.187 |
| Stage I hypertension-increasing | 1.78 (1.20, 2.71) | 0.005 | 1.81 (1.15, 2.90) | 0.012 | 1.65 (1.04, 2.67) | 0.036 | 1.54 (0.97, 2.50) | 0.073 |
| Stage II hypertension-increasing | 2.53 (1.47, 4.37) | 0.001 | 2.16 (1.17, 3.99) | 0.014 | 1.80 (0.95, 3.43) | 0.071 | 1.53 (0.80, 2.95) | 0.198 |
| **Females, n = 2316** |  |  |  |  |  |  |  |  |
| Normotensive-stable | Ref |  | Ref |  | Ref |  | Ref |  |
| Prehypertension-stable | 1.32 (1.04, 1.70) | 0.025 | 1.49 (1.14, 1.94) | 0.003 | 1.52 (1.17, 1.99) | 0.002 | 1.46 (1.12, 1.92) | 0.005 |
| Stage I hypertension-increasing | 1.49 (1.10, 2.00) | 0.009 | 1.67 (1.18, 2.35) | 0.003 | 1.75 (1.22, 2.50) | 0.002 | 1.58 (1.10, 2.28) | 0.013 |
| Stage II hypertension-increasing | 1.73 (1.08, 2.73) | 0.021 | 1.89 (1.10, 3.21) | 0.019 | 2.11 (1.16, 3.79) | 0.013 | 1.74 (0.95, 3.16) | 0.070 |

Model 1: Unadjusted.

Model 2: Adjusted for age, gender (only for total), follow-up years, baseline SBP and BMI.

Model 3: Adjusted for variables in Model 2 +smoking, alcohol drinking, physical-activity, energy intake and antihypertensive drugs use.

Model 4: Adjusted for variables in Model 3 +BMI trajectory groups.

BMI, body mass index; SBP, systolic blood pressure.

**Table S9**. Odds ratios and 95% CIs for joint trajectories of SBP and BMI groups for incident prediabetes.

|  |  | Model 1 |  | Model 2 |  | Model 3 |  |
| --- | --- | --- | --- | --- | --- | --- | --- |
|  | % (n/N) * | OR (95%*CI*) | *P* | OR (95%*CI*) | *P* | OR (95%*CI*) | *P* |
| **BMI normal-stable** |  |  |  |  |  |  |  |
| Normotensive-stable | 17.5 (95/543) | Ref |  | Ref |  | Ref |  |
| Prehypertension-stable | 21.2 (270/1273) | 1.27 (0.98, 1.65) | 0.071 | 1.34 (1.02, 1.77) | 0.035 | 1.36 (1.03, 1.79) | 0.030 |
| Stage I hypertension-increasing | 27.3 (114/418) | 1.77 (1.30, 2.41) | <0.001 | 1.87 (1.33, 2.64) | <0.001 | 1.86 (1.31, 2.63) | <0.001 |
| Stage II hypertension-increasing | 27.3 (12/44) | 1.77 (0.85, 3.47) | 0.110 | 1.90 (0.87, 3.90) | 0.091 | 1.77 (0.81, 3.69) | 0.137 |
| **BMI overweight / obesity increasing** |  |  |  |  |  |  |  |
| Normotensive-stable | 24.8 (50/202) | 1.55 (1.05, 2.28) | 0.027 | 1.44 (0.95, 2.17) | 0.085 | 1.44 (0.95, 2.17) | 0.086 |
| Prehypertension-stable | 27.9 (280/1003) | 1.83 (1.41, 2.38) | <0.001 | 1.93 (1.44, 2.61) | <0.001 | 1.94 (1.44, 2.62) | <0.001 |
| Stage I hypertension-increasing | 27.9 (202/725) | 1.82 (1.39, 2.40) | <0.001 | 2.07 (1.49, 2.88) | <0.001 | 2.03 (1.45, 2.84) | <0.001 |
| Stage II hypertension-increasing | 34.3 (58/169) | 2.46 (1.67, 3.62) | <0.001 | 2.44 (1.55, 3.81) | <0.001 | 2.38 (1.47, 3.84) | <0.001 |

Model 1: Unadjusted.

Model 2: Adjusted for age, gender, follow-up years, baseline SBP and BMI.

Model 3: Adjusted for variables in Model 2 +smoking, alcohol drinking, physical-activity, energy intake and antihypertensive drugs use.

BMI, body mass index; SBP, systolic blood pressure. *Prediabetes event number divided by total number in each group.
